# Interleukin-1 Receptor-Associated Kinase 1 in Cancer Metastasis and Therapeutic Resistance: Mechanistic Insights and Translational Advances

**DOI:** 10.3390/cells13201690

**Published:** 2024-10-12

**Authors:** Mariana K. Najjar, Munazza S. Khan, Chuling Zhuang, Ankush Chandra, Hui-Wen Lo

**Affiliations:** 1 Vivian L. Smith Department of Neurosurgery, McGovern Medical School, The University of Texas Health Science Center at Houston, Houston, TX 77030, USA; mariana.k.najjar@uth.tmc.edu (M.K.N.); munazza.samar.khan@uth.tmc.edu (M.S.K.); chuling.zhuang@uth.tmc.edu (C.Z.); ankush.chandra@uth.tmc.edu (A.C.); 2 Graduate School of Biomedical Sciences, The University of Texas Health Science Center at Houston, Houston, TX 77030, USA; 3 Department of Integrative Biology and Pharmacology, McGovern Medical School, The University of Texas Health Science Center at Houston, Houston, TX 77030, USA

**Keywords:** IRAK1, metastasis, TLR/IL-1R axis, therapeutic resistance, IRAK1 inhibitors

## Abstract

Interleukin-1 Receptor Associated Kinase 1 (IRAK1) is a serine/threonine kinase that plays a critical role as a signaling transducer of the activated Toll-like receptor (TLR)/Interleukin-1 receptor (IL-1R) signaling pathway in both immune cells and cancer cells. Upon hyperphosphorylation by IRAK4, IRAK1 forms a complex with TRAF6, which results in the eventual activation of the NF-κB and MAPK pathways. IRAK1 can translocate to the nucleus where it phosphorylates STAT3 transcription factor, leading to enhanced IL-10 gene expression. In immune cells, activated IRAK1 coordinates innate immunity against pathogens and mediates inflammatory responses. In cancer cells, IRAK1 is frequently activated, and the activation is linked to the progression and therapeutic resistance of various types of cancers. Consequently, IRAK1 is considered a promising cancer drug target and IRAK1 inhibitors have been developed and evaluated preclinically and clinically. This is a comprehensive review that summarizes the roles of IRAK1 in regulating metastasis-related signaling pathways of importance to cancer cell proliferation, cancer stem cells, and dissemination. This review also covers the significance of IRAK1 in mediating cancer resistance to therapy and the underlying molecular mechanisms, including the evasion of apoptosis and maintenance of an inflammatory tumor microenvironment. Finally, we provide timely updates on the development of IRAK1-targeted therapy for human cancers.

## 1. Introduction

Cancer is the second most common cause of death in the United States following heart disease [1,2]. The majority of cancer-associated deaths and treatment failures are caused by the formation of distant metastases [3,4,5]. Development of metastases is mediated by complex processes that involve different cell types, the surrounding microenvironment, and multiple key signaling pathways [6,7]. Notably, over the past two decades, clear evidence has shown that the inflammatory profile of the tumor microenvironment (TME) significantly contributes to cancer progression, metastasis formation, and therapeutic resistance [8,9,10,11,12].

One of the major contributors to inflammation is innate immune signaling, which is activated by the detection of different cytokines or pathogens through several surface receptors, including pattern recognition receptors (PRRs) [13,14,15,16]. Members of the TLR/IL-1R superfamily are some of the most upstream receptors that, upon the recognition of their cognate ligands, undergo homo- or heterodimerization and initiate a downstream signaling cascade [17]. This process involves the recruitment of adaptor proteins, which in turn activate members of the IRAK family of proteins, leading to the regulation of various inflammatory genes that play a central role in innate immunity [13,18,19,20]. TLR/IL-1R signaling plays an important role in fostering a pro-inflammatory TME. Numerous studies have demonstrated this pathway’s involvement in the production of pro-inflammatory cytokines and chemokines, growth factors, and anti-apoptotic proteins in inflammatory cells within the tumors, all of which contribute to tumor progression and chemoresistance [17,21,22,23,24,25].

IRAKs are a unique family of serine/threonine kinases that consist of four members— IRAK1, IRAK2, IRAK3 (also known as IRAK-M), and IRAK4. Of the four members, human IRAK1, IRAK2, and IRAK4 are ubiquitously expressed while IRAK-M is only induced in monocytes and macrophages [26,27,28]. The structure of IRAK proteins is highly conserved among all four members and consists of an N-terminal death domain (DD), a ProST domain (rich in proline, serine, and threonine residues), and a kinase/pseudokinase domain (KD). IRAK1, IRAK2, and IRAK-M also contain a C-terminal TRAF6 binding motif (TBM) [29,30]. Although all IRAKs are classified as serine/threonine kinases and share a kinase-like domain, only IRAK1 and IRAK4 exhibit verified kinase activity [31]. IRAK-M and IRAK2 are considered as pseudokinases or possible pseudokinases, and are characterized by the substitution of the aspartic acid residue in the KD of IRAK1 and IRAK4 with serine or asparagine, respectively. Despite lacking the key aspartate residue required for the kinase activity, IRAK2 contains an ATP-binding pocket with a lysine residue, allowing it to function as an atypical kinase [32].

**Figure 1:**
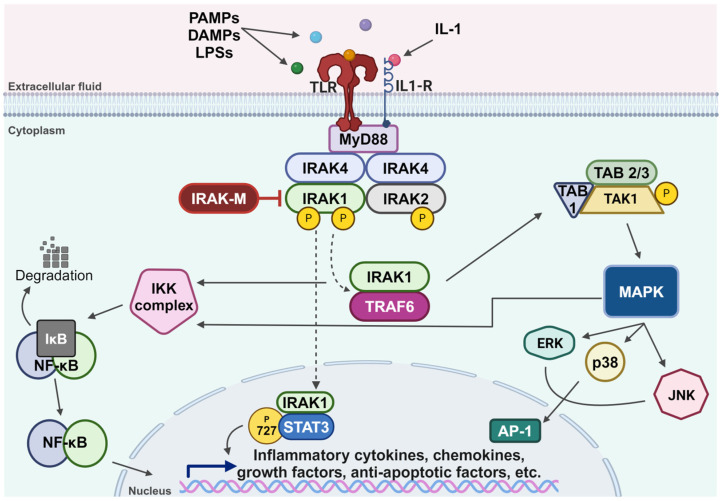
An overview of the TLR/IL-1R signaling pathway. The TLR/IL-1R pathway is activated following TLR or IL-1R binding to their respective ligands, including PAMPs, DAMPs, LPSs, or cytokines from the IL-1 family. This binding triggers the recruitment of MyD88 and assembly of the myddosome complex, comprising MyD88, IRAK4, IRAK2, and IRAK1. IRAK4 phosphorylates IRAK1, initiating an auto-phosphorylation cascade that results in hyperphosphorylated IRAK1. Hyperphosphorylated IRAK1 then dissociates from the myddosome complex and associates with TRAF6. IRAK-M functions to inhibit the dissociation of IRAK1 from the myddosome. The interaction between IRAK1 and TRAF6 activates the IKK complex, leading to the degradation of IκB, which releases NF-κB for nuclear translocation and transcriptional activity. Additionally, IRAK1 and TRAF6 interaction leads to the assembly of the catalytically active TAK1-TAB complex, which activates the MAPK pathway. In addition to activating the IKK complex, MAPK activates downstream effectors ERK, p38, and JNK, all of which are involved in the transcriptional activation of several inflammatory cytokines through AP-1. Moreover, IRAK1 translocates to the nucleus, where it phosphorylates STAT3 at the serine 727 residue, promoting the IL-10 gene activation. Created with BioRender.com.

Members of the IRAK family of kinases are key regulators of the TLR/IL-1R signaling pathway, which is activated following binding to the receptors’ cognate ligands, such as protein-associated molecular patterns (PAMPs), damage-associated molecular patterns (DAMPs), lipopolysaccharides (LPSs), and multiple members of the IL-1 family of cytokines [13,17,18,19,20,33,34] (Figure 1). This activation triggers an inflammatory response mediated by the assembly of a multiprotein complex known as myddosome, composed of Myeloid Differentiation primary response protein 88 (MyD88), IRAK4, and IRAK2. Within this complex, IRAK1 is activated through IRAK4-dependent phosphorylation, followed by autophosphorylation that leads to its hyperphosphorylation [35,36,37,38,39]. Upon hyperphosphorylation, IRAK1 is released from the myddosome complex and associates with TNF Receptor-Associated Factor 6 (TRAF6) protein, an E3 ubiquitin ligase. This interaction results in the activation of the IκB Kinase (IKK) complex, leading to the degradation of IkappaB and eventual nuclear translocation of Nuclear Factor-kappa B (NF-κB) [40]. The IRAK1 and TRAF6 complex can also lead to the assembly of the catalytically active Transforming Growth Factor beta-Activated Kinase 1-TAK1-Binding protein (TAK1-TAB) complex, which in turn activates the MAPK pathway and the IKK complex [41]. Additionally, phosphorylated IRAK1 can translocate to the nucleus, where it phosphorylates STAT3 at its serine 727 residue, subsequently promoting IL-10 gene expression [42]. IRAK1 protein gets degraded within an hour of its activation, correlating with a rapid decline in its activity; however, IRAK2 protein remains stable and can sustain TLR/IL-1R signaling for a longer duration [32,43]. While IRAK1, IRAK2, and IRAK4 function to amplify the signaling within the TLR/IL-1R axis, IRAK-M serves as a negative regulator of the pathway [44]. It inhibits the dissociation of IRAK1 from the myddosome complex, thereby preventing its association with TRAF6 [44].

The dysregulation of the TLR/IL-1R pathways is implicated in the pathogenesis of many diseases [45]. Recent studies have highlighted the role of IRAKs in the pathophysiology of cancers, metabolic disorders, and cardiovascular and inflammatory conditions [45,46,47,48]. Given the critical role IRAK1 plays in the activation of this pathway, research efforts have focused on its involvement in the progression of various cancer types. These studies led to the discovery of many potential therapeutic agents, offering promising benefits across different types of cancer [49,50]. While IRAK1 has been implicated in a variety of cellular processes beyond cancer, this review is specifically focused on its role in cancer metastasis and its associated mechanisms. Other significant aspects of IRAK1 exist but are not covered in this review.

## 2. IRAK1 in Cancer

IRAK1 plays a pivotal role as one of the two putative kinases that are responsible for converging downstream signaling of the TLR/IL-1R signaling pathways and is increasingly recognized for its involvement in aberrant TLR/IL-1R signaling in cancer biology. A study by Pilarsky et al. in 2004 first reported that *IRAK1* gene expression is elevated in most cancer types, with the exception of thyroid carcinoma (THCA) [51]. These findings were later corroborated by Lui et al., who conducted a comprehensive analysis of IRAK1 expression across various types of cancer [28]. They reported that the mRNA levels of *IRAK1* are upregulated in nearly all cancer types, with the notable exceptions of THCA and acute myeloid leukemia (LAML), where its expression is much lower compared to normal tissues. IRAK1 has been implicated in various malignancies, contributing to tumor progression, metastases formation, and treatment resistance. The involvement of IRAK1 in these roles has been extensively studied in both cellular and animal models using different tools, such as genetic overexpression, knockouts, pharmacological inhibitions, and endogenous downregulation, to elucidate its mechanism and aid in the discovery of potent inhibitors. Recent publications have summarized the role of IRAK1 in different types of cancers, while emphasizing its significance as a therapeutic target [49,50,52]. This review aims to elucidate the mechanistic role of IRAK1 in cancer metastasis, with a focus on its involvement in mediating therapeutic resistance. Additionally, this review will highlight recent advancements in the development of IRAK1 inhibitors and their clinical implications.

### 2.1. IRAK1 Genetic Alterations in Cancer

In a 2022 study, a comprehensive analysis was conducted to determine *IRAK1* alterations across various types of cancer [28]. The researchers identified amplification as the most common DNA alteration, likely contributing to the high IRAK1 expression observed in tumor tissues. They also identified two specific *IRAK1* mutations situated in the KD, Q180H/*, and G224E, both of which are associated with improved prognoses and appear to diminish the role of IRAK1 in cancer progression [28]. A study focused on primary effusion lymphoma (PEL) reported a missense mutation in *IRAK1*, resulting in a Phe196Ser substitution that renders IRAK1 constitutively active, marking it as the main driver for Kaposi sarcoma herpesvirus lymphoma [53]. In a separate study, Li et al. induced a point mutation (K239A) in the ATP-binding site of IRAK1 and reported that mutant IRAK1 remained active and was capable of inducing NF-κB activation, suggesting that IRAK1 can be activated by different kinases [54]. In 2001, Jensen et al. identified an alternatively spliced variant of IRAK1, IRAK1b, which is kinase-inactive but functionally active and highly stable following IL-1 stimulation [55]. Collectively, these findings indicate that IRAK1 functions are not strictly dependent on its catalytic activity, underscoring its significance in cancer development and highlighting the importance of developing IRAK1 inhibitors.

### 2.2. Regulation of IRAK1 Expression by miRNAs

MicroRNAs (miRs) are small non-coding regulatory RNAs that play a significant role in the regulation of gene expression via controlling protein synthesis at the post-transcriptional level [56,57]. Analyses of the miR-146a promoter revealed it as an NF-κB-dependent gene and predicted its base-pairing potential with IRAK1 [58,59]. MiR-146a was found to target mRNAs encoding for IRAK1 whose function is associated with tumorigenesis [60,61,62,63,64,65,66].

One of the studies highlighting the regulatory effect miR-146a-5p has on IRAK1 following binding was conducted by Long et al. in 2018 on breast cancer cells [60]. They discovered that the expression of IRAK1 was downregulated following direct binding of miR-146a-5p to *IRAK1*’s 3’-untranslated region, leading to the suppression of migration and invasion in breast cancer cells. Additionally, they revealed that restoring IRAK1 reversed these effects, resulting in a more aggressive phenotype [60]. Their findings were supported by another study that identified miR-146a/b as negative regulators of IRAK1, leading to the suppression of NF-κB activity and reduction in the metastatic potential of breast cancer [61]. Similar findings reported that miR-146a, through the inhibition of *IRAK1*, retards tumor progression in lung adenocarcinoma cells, and the invasive potential of cervical and colorectal cancer (CRC) cells [63,64]. A more recent study found that miR-146a-5p is deficient in extracellular vesicles (EVs) released by glioma-associated macrophages (GAMs). The introduction of a miR-146a-5p mimic led to the downregulation of IRAK1 in glioma cells, which in turn significantly reduced their invasive potential [62]. Additional studies on tumor-associated macrophages (TAMs) and TAM-derived exosomes revealed that miR-192-5p limits the growth and progression of endometrial cancer (EC) through the inhibition of the IRAK1/NF-κB signaling pathway [67].

Conversely, a 2016 study revealed that IRAK1 is associated with less cell aggressiveness in papillary thyroid carcinoma (PTC) [65]. They demonstrated a reduction in the expression of IRAK1 following binding of miR-146b-5p led to higher rates of metastasis and worse outcomes [65]. Similar findings were reported in oral squamous cell carcinoma (OSCC) where exogenous miR-146a expression increased the tumorigenicity and metastasis *in vivo* [66]. These findings indicate that the expression levels of IRAK1 are positively correlated with better outcomes in PTC and OSCC [65,66].

In addition to miR-146, a Kaposi’s sarcoma-associated herpesvirus (KSHV) miRNA, miR-K12-9, has been shown to inhibit IRAK1 expression and decrease NF-κB activity, resulting in reduced inflammatory cytokine expression during viral infection [68]. This mechanism likely enhances the virus’s ability to successfully infect the host and promotes progression to Kaposi’s sarcoma or other types of lymphoma.

These studies underscore the importance of further research promoting the use of the above-mentioned miRNA in predicting cancer severity, guiding treatment selection, and evaluating responsiveness to therapies targeting IRAK1.

## 3. IRAK1 in Cancer Metastasis

**Figure 2:**
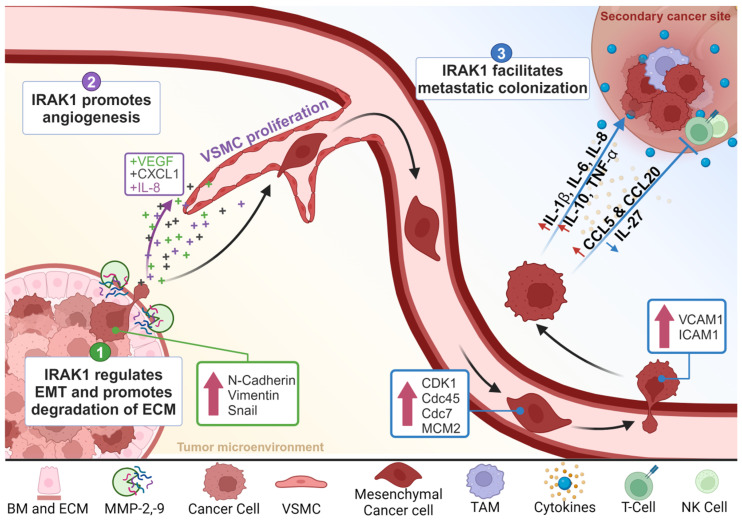
Roles of IRAK1 in the metastatic cascade. (1) IRAK1 regulates the epithelial–mesenchymal transition. It upregulates the expression of mesenchymal markers (N-cadherin, vimentin, and snail), along with endopeptidases (MMP-2 and MMP-9), for degradation and remodeling of the extracellular matrix (ECM). (2) IRAK1 promotes angiogenesis. It elevates the expression of pro-angiogenic factors (VEGF, CXCL1, and IL-8), and is involved in vascular smooth muscle cell (VSMC) proliferation. (3) IRAK1 facilitates metastatic colonization. It promotes survival through releasing pro-growth cytokines and activating mitotic cell cycle-related factors (CDK1 and Cdc45) and cell division pathway factors (Cdc7 and MCM2). It facilitates the extravasation of circulating tumor cells (CTCs) through upregulating the expression of adhesion molecules (VCAM1 and ICAM1). It supports cell colonization at secondary sites by modulating the TME through the release of pro-growth and pro-survival cytokines, and the promotion of immune evasion. Created with BioRender.com.

Inflammatory dysregulation is associated with malignant progression and metastasis formation in most types of cancers [69,70]. IRAK1 is converged upon by multiple oncogenic signaling axes and is increasingly recognized as a pivotal player in the complex process of cancer metastasis. While the role of IRAK1 in the context of innate immunity is well established, emerging research highlights its involvement in the various stages of the metastatic cascade, including cancer cell migration, invasion, and finally colonization in different metastatic sites, as summarized in Figure 2 and Table 1.

### 3.1. IRAK1 in Cancer Cell Migration and Invasion

The ability of cancer cells to undergo migration and invasion is essential for successful metastasis, which requires tumor cells to detach from the primary tumor, breach the basement membrane, penetrate the ECM, and enter the bloodstream or lymphatic system [5,6,7,101]. IRAK1 has been implicated in promoting various aspects of these invasive phenotypes across several cancer types [60,62,63,64,65,66,71,72,73,74,75,80,81,102,103].

#### 3.1.1. IRAK1 Regulates EMT

One of the mechanisms by which IRAK1 facilitates tumor migration and invasion is through the regulation of the EMT process [62,71,73,74,75]. During this process, epithelial cells lose their polarity, which normally allows them to interact with the basement membrane, and acquire mesenchymal characteristics that enhance their migratory capacity [104,105]. 

In 2020, Chen and colleagues elucidated a mechanism by which IRAK1 promotes metastasis in hepatocellular carcinoma (HCC) through the activation of the NLRP3/MAPKs/IL-1β pathway [71]. Their study demonstrated that the knockdown of IRAK1 led to a marked reduction in the migration and invasion capabilities of several HCC cell lines, an effect they attributed to the suppression of NLRP3 inflammasome activity. Additionally, they investigated the role of IRAK1 in the EMT process and found that IRAK1 knockdown significantly upregulated the protein expression levels of the epithelial marker E-cadherin and downregulated the protein expression levels of the mesenchymal markers N-cadherin and vimentin. These effects were also linked to the suppression of NLRP3 inflammasome activity following IRAK1 knockdown [71]. The involvement of IRAK1 in the activation of NLRP3 inflammasomes and its effect on the migration and invasion of HCC were also highlighted by another study in 2022 [72]. Moreover, in 2021, a study on CRC by Feng et al. demonstrated that, following stimulation with IL-1β, the pharmacological inhibition of IRAK1 decreased cell migration *in vitro* and resulted in a significant increase in the epithelial marker E-cadherin levels and decrease in mesenchymal marker N-cadherin, vimentin, and Snail levels. These effects were also observed *in vivo* using their colitis-induced tumorigenesis mouse model. The authors linked these changes in the expression of protein to the decreased activation of NF-κB [73]. Further supporting these findings, a 2022 study reported similar effects in low-grade glioma (LGG) where the knockdown of IRAK1 resulted in higher E-cadherin expression and lower expression levels of N-cadherin and vimentin *in vitro* [74]. Additionally, studies on glioma cells reported similar findings where IRAK1 knockdown led to the reduction in migration and invasion, alongside an increase in E-cadherin expression and a decrease in N-cadherin and vimentin levels [62,75]. 

Collectively, these studies describe the involvement of IRAK1 in the activation of downstream pathways, including NF-κB and MAPKs/NLRP3/IL-1β, which promote the production of mesenchymal markers that induce a shift in the cellular phenotype of cancer cells. This shift is crucial for the initial stages of metastasis, as it endows cancer cells with the plasticity needed to invade and migrate [5,6,7,101,104,105].

#### 3.1.2. IRAK1 Activates MMPs

Another mechanism by which IRAK1 facilitates cancer cell migration and invasion is through the activation of MMPs, a family of endopeptidases that degrade proteins within the ECM. MMPs break down the ECM into smaller components, thereby facilitating tumor invasion [79]. Particularly, IRAK1 has been reported to enhance the expression levels of MMP-2 and MMP-9, the two most prominent MMPs involved in the process of metastasis [60,76,77,78,79,80,81] 

In 2021, a study by Lin et al. revealed that IRAK1 enhances the expression of both MMP-2 and MMP-9 in T-cell acute lymphoblastic leukemia (T-ALL) through the activation of NF-κB, specifically NF-κB p65 activity [80]. The study demonstrated that in the presence of IRAK1, the knockdown of p65 failed to induce an increase in MMP-2 and MMP-9 expression, further underscoring the critical role of IRAK1 in driving the expression of these MMPs through the downstream NF-κB p65 activation. Additionally, the inhibition of IRAK1 led to a reduction in MMP-2 and MMP-9 expression levels, highlighting the importance of IRAK1 in regulating these proteases [80]. 

Similarly, a study on endometrial carcinoma found that silencing IRAK1 inhibited the expression of migration- and invasion-related proteins MMP-2 and MMP-9 [81]. A separate study on breast cancer also demonstrated that the inhibition of IRAK1 led to a reduction in MMP-2 and MMP-9 protein expression, further supporting the role of IRAK1 in the promotion of cancer cell metastasis [60]. Additionally, a study on glioma cells reported similar findings where IRAK1 knockdown led to the reduction in MMP-2 protein levels [62].

The upregulation of MMPs by IRAK1 involves the activation of downstream signaling pathways such as NF-κB and AP-1, both of which are critical for the transcriptional activation of MMP genes. This proteolytic degradation of ECM not only promotes invasion but also creates a favorable microenvironment for tumor progression and metastasis [76,77,78,79,80].

### 3.2. IRAK1 in Angiogenesis

Angiogenesis, the formation of new blood vessels, is a key component of the metastatic pathway. It provides nutrients and oxygen to proliferating cancer cells and offers a route for disseminating cells to enter the circulation [106,107]. IRAK1 has been implicated in the regulation of angiogenesis through multiple mechanisms [82,83,108,109,110].

#### 3.2.1. IRAK1 Increases the Expression of Pro-Angiogenic Molecules

The activation of the TLR/IL-1R axis triggers IRAK1 propagating downstream NF-κB activation and the subsequent expression of various pro-angiogenic factors essential for tumor growth and metastasis such as VEGF, CXCL1, and IL-8. These findings were further validated in a study on melanoma cell lines, which also demonstrated a reversal of this effect following IRAK1 inhibition [82]. Supporting these results, another study reported a reduction in the MVD of tumors resected from lenvatinib-treated mice following treatment with IRAK1 inhibitors [83].

The contribution of IRAK1 in angiogenesis has also been extensively studied in the context of wound healing and autoimmune diseases, outside of cancer. In these settings, the involvement of IRAK1 in the regulation of angiogenic molecules is critical for both the promotion of tissue repair and the pathogenesis of autoimmune conditions [84,108,109,110].

#### 3.2.2. IRAK1 Is Involved in Vascular Smooth Muscle Cell Proliferation

VSMCs are essential for the formation of new blood vessels. In a 2015 study, Jain et al. identified a novel function for IRAK1 in promoting VSMC proliferation through the activation of ERK1/2, which subsequently leads to the reduction in p27^Kip1^, a CDK inhibitor. The study elucidated the IRAK1-dependent nature of ERK1/2 activation using both IRAK1 inhibitors and knockdown models. Additionally, it was confirmed that the reduction in p27^Kip1^ was mediated by ERK1/2 activation as the use of an ERK inhibitor restored p27^Kip1^ levels without affecting IRAK1 activation. Furthermore, the study revealed that IRAK1 activation is dependent on both TLR and PKC-ε pathways [84].

### 3.3. IRAK1 in Metastatic Colonization

Once cancer cells disseminate through the bloodstream or lymphatic system, they must survive in the circulation, extravasate into distant tissues, and establish secondary tumors, all of which are steps collectively known as metastatic colonization [111]. IRAK1 has been found to be associated with each one of these steps, further solidifying its role in metastasis.

#### 3.3.1. IRAK1 Promotes Survival of CTCs

CTCs encounter various stresses in the circulation and must overcome these challenges to survive. IRAK1 plays a pivotal role in supporting the survival of CTCs by resisting apoptosis, primarily through the activation of NF-κB and subsequent pro-growth and survival signaling. A study conducted on the activated B-cell-like (ABC) subtype of diffuse large B-cell lymphoma (DLBCL) highlighted the involvement of IRAK1 in the survival of ABC DLBCL cell lines via NF-κB signaling, the JAK kinase activation of STAT3, and the secretion of IL-6, IL-10, and IFN-β, all of which contribute to cell survival. This study also underscores the importance of the upstream adaptor protein MyD88 for this function. Correspondingly, the use of an IRAK1 inhibitor led to increased apoptosis, indicating the importance of IRAK1 in regulating the expression of these pro-growth cytokines [85].

Another mechanism by which IRAK1 acts to promote the survival of CTCs is through the activation of mitotic cell cycle and cell division pathways [81]. This was illustrated in a study on EC, which reported a positive correlation between IRAK1 and the expression of these pathways in patient tissue samples. The findings were further validated in EC cells, where silencing of IRAK1 induced cell cycle arrest and apoptosis, and suppressed the expression of mitotic cell cycle-related factors (CDK1 and Cdc45) and cell division pathway factors (Cdc7 and MCM2) [81].

A recent study identified an IRAK1-induced apoptosis resistance mechanism that operates independently of the canonical TLR/IL-1R signaling [91]. Li et al. demonstrated that radiation-induced double-stranded DNA breaks activate the DNA damage response kinase ATR, leading to the activation and nuclear translocation of IRAK1 by Pellino ligases. Once in the nucleus, IRAK1 bound to and inhibited a proapoptotic complex, thereby promoting cell survival [91]. 

Overall, numerous studies that utilize IRAK1 inhibitors, knockdown, or overexpression models have consistently highlighted the role of IRAK1 in the inhibition of cancer cell apoptosis and promotion of cell survival [74,86,87,88,89,90].

#### 3.3.2. IRAK1 Facilitates Extravasation of CTCs

Upon reaching distant organs, tumor cells must first adhere to or roll or slide along the endothelial cells lining the blood vessels, and eventually form a tight bond that allows them to move into tissues [112,113]. IRAK1 facilitates this process through the promotion of adhesion molecule (VCAM-1 and ICAM-1) expression, which enables tumor cells to adhere to the endothelium [92,94]. Several studies have shown that IL-1β stimulates IRAK1 activation, leading to the subsequent NF-κB activation and enhanced expression of VCAM-1 and ICAM-1 [92,93,94]. This increase in adhesion molecule expression was abrogated following treatment with IRAK1 inhibitors [94].

#### 3.3.3. IRAK1 Supports Cancer Cell Colonization at Secondary Sites

Colonization is the final step cancer cells must take to establish metastasis at the secondary site. This process involves cancer cell survival, latency, and growth [5,101,111,114]. In addition to the activation of pro-survival and pro-growth cytokines and resistance to apoptosis, IRAK1 induces the secretion of multiple factors that suppress local immune responses, thereby reconstructing a favorable niche that allows metastatic cells to thrive.

In 2021, a study by Cai et al. on stem cell leukemia/lymphoma syndrome (SCLL) reported that IRAK1 expression regulates IFN-γ signaling, which results in increased levels of myeloid-derived suppressor cells (MDSCs) and a corresponding decrease in CD4+/CD8+ T-cell levels, thereby facilitating immune evasion both *in vitro* and *in vivo*. Notably, the inhibition of IRAK1 reversed all these effects [95]. 

Additionally, IRAK1 has been found to reduce the production of IL-27, an antitumor cytokine, in a STAT1-dependant mechanism in macrophages [96]. IL-27 is crucial for enhancing Th1, CD8^+^ CTL, and NK cell responses, all of which are involved in the antitumor immune response [96,115,116]. Elevated IRAK1 expression results in decreased IL-27 levels, thereby weakening antitumor immunity at the metastatic site. 

Another mechanism through which IRAK1 promotes immune evasion is highlighted in a study by Sanmiguel et al.; they identified a direct link between IRAK1 and the regulation of T-cell-targeting chemokine (CCL5 and CCL20) production, both of which are reported to influence tumor progression [97,117,118]. The study reported that the overexpression of IRAK1 led to an increased constitutive and cytokine-induced production of CCL5 and CCL20, while the reverse was observed with the use of IRAK1 inhibitors and IRAK1 knockdown [97].

Moreover, IRAK1 activation in TAMs is reported to promote a STAT3-dependent tumoricidal to tumor-promoting shift towards the M2 polarization of macrophages [119]. M2 macrophages are characterized as anti-inflammatory macrophages that, through the production of immunosuppressive cytokines and growth factors, support tumor growth and metastasis and participate in immune suppression [98].

### 3.4. IRAK1 in the TME

During every stage of metastasis, CTCs are exposed to a complex ecosystem known as the TME, which consists of various cell types and ECM components. IRAK1 modulates several aspects of the TME, further facilitating cancer metastasis.

One of the primary ways IRAK1 influences the TME is through the secretion of pro-inflammatory cytokines such as IL-1β, IL-6, IL-8, IL-10, and TNF-α, all of which can contribute to a pro-tumorigenic microenvironment. In a study by Mahmoud et al., the inhibition of IRAK1 significantly reduced the secretion of these cytokines, which was accompanied by suppressed cancer cell growth [99].

In addition to promoting a pro-tumorigenic microenvironment, IRAK-1 participates in the crosstalk between cancer cells and CAFs in the tumor microenvironment [100]. A co-culture study on lingual squamous cell carcinoma identified that IL-1β released by cancer cells leads to an upregulation of IL-1R and increased activation of IRAK-1 in CAFs. This triggers the activation of NF-κB and the transcription of *IL-6*, *Cox-2*, *BDNF*, and *IRF-1*, fostering tumor progression [100].

Therefore, IRAK1 has been recognized as a critical metastasis-promoting target, with various therapeutic strategies needed to focus on modulating or inhibiting its activity. These approaches hold significant promise for the prevention of cancer progression. 

## 4. IRAK1 in Therapeutic Resistance

Together with promoting metastasis, IRAK1 drives therapeutic resistance in different types of cancers. Presently, drug resistance remains to pose a major challenge, contributing to treatment failures and poor patient outcomes. The primary mechanism through which IRAK1 promotes therapeutic resistance is through the activation of its downstream effectors including NF-κB, MAPK, and STAT3.

### 4.1. IRAK1 Induces Resistance to Therapeutic Agents

Traditional chemotherapies and targeted therapies have revolutionized cancer treatment. However, the common issue of therapeutic resistance remains a significant challenge [120,121]. IRAK1, with its prominent role in promoting pro-survival factors, significantly undermines the effectiveness of these agents by counteracting their mechanisms of action.

A study by Wee et al. on breast cancer reported strong IRAK1 activation following paclitaxel treatment [86]. This activation was associated with an increased expression of inflammatory cytokines and subsequent enrichment of cancer stem cells (CSCs), all of which indicate that IRAK1 contributes to acquired resistance to paclitaxel treatment. They reported that the underlying mechanism for this resistance is partly attributed to the activation of the p38-MCL1 pro-survival pathway, which was reversed following treatment with IRAK1 inhibitors [86]. These findings were further supported by other studies that utilized miR-146a or GPT, one of the main active components in *Panax ginseng,* to restore the sensitivity of breast cancer cells to paclitaxel treatment [122,123]. 

Additionally, IRAK1 is also involved in a functional regulatory circuit with products of chromosome 1q21.3 amplification, enriched in breast cancer, leading to breast cancer recurrence. The use of IRAK1 inhibitors disrupts this feedback loop [124].

The role of IRAK1 in the activation of survival pathways leading to therapeutic resistance has been implicated in HER2-enriched breast cancer [125,126]. A study by Liu et al. correlated the overexpression of HER2 and its stimulation of an inflammatory milieu to the induction of a feedforward activation loop of IL-1α and IL-6 [125]. This is mediated through the activation of the TLR/IL-1R pathway that subsequently sustains NF-κB and STAT3 pathways through the activation of IRAK1. The hyperactivation of this pathway aids in the generation and maintenance of a treatment-resistant breast CSC population and it was reversed following treatment with IRAK1 inhibitors [125].

Moreover, IRAK1 induces chemoresistance in nasopharyngeal carcinoma through the IRAK1–S100A9 axis [127]. NC samples exhibit increased levels of active IRAK1, which enhances the *S100A9* expression. The overexpression of *S100A9* is positively correlated with paclitaxel resistance in nasopharyngeal carcinoma. Through *in vitro* and *in vivo* studies, Liu et al. reported that an IRAK1 blockade reversed the IRAK1-S100A9-induced paclitaxel resistance [127].

In 2018, Cheng et al. identified an IRAK1-dependent mechanism underlying doxorubicin and sorafenib resistance in HCC [128]. Using *in vitro* and *in vivo* studies, they demonstrated that high levels of IRAK1 in HCC enhance the expression of AKR1B10, a downstream effector of AP-1 and a main regulator of tumor-initiating cells (TICs), thereby promoting doxorubicin or sorafenib resistance. The pharmacological inhibition of IRAK1 was shown to suppress TIC populations and sensitized the cells to doxorubicin and sorafenib treatment [128].

Melgar et al. identified a causation relationship between the IRAK1-mediated activation of the innate inflammatory pathway and resistance to FLT3 inhibitor in FLT3-mutated AML [129]. Furthermore, IRAK1 is reported to contribute to chemotherapy resistance through the activation of NF-κB, which in turn upregulates the expression of P-glycoprotein (P-gp), a drug efflux transporter, reducing the intracellular concentration of chemotherapies and diminishing their efficacy [130]. This mechanism is particularly significant in AML patients, where IRAK1 overexpression has been linked to resistance against anthracycline [131]. In MDS/AML patients, IRAK4 has been recognized as a therapeutic target and IRAK4 inhibitors have advanced clinical trials. However, resistance to IRAK4 inhibitors has emerged in these patients, which Bennett et al. attributed to a non-canonical compensatory mechanism by IRAK1. This finding underscores the need for dual IRAK1/4 inhibitors for treatment of MDS/AML patients to overcome resistance and improve therapeutic outcomes [52].

A study on non-small cell lung cancer (NSCLC) therapeutic resistance identified a role of miR-146b-5p in downregulating IRAK1, thereby enhancing the sensitivity to EGFR tyrosine kinase inhibitors (TKIs). Restoring the expression of *IRAK1* counteracted the effects of miR-146b-5p on EGFR TKI sensitivity, emphasizing the direct involvement of IRAK1 in mediating this therapeutic resistance [132].

### 4.2. IRAK1 Induces Resistance to Radiation Therapy

Radiation therapy is one of the standards of care treatment modalities in various types of cancers, particularly in localized tumors. Radiotherapy primarily works through the induction of DNA damage in cancer cells, leading to their death. Resistance to radiotherapy remains a clinical challenge that often leads to incomplete tumor eradication and recurrence [133,134]. Several recent studies uncovered a role for IRAK1 in mediating resistance to radiation therapy [75,91,135,136,137].

A 2024 study on cervical cancer identified a negative correlation between IRAK1 expression and the efficacy of radiotherapy [137]. They attributed these findings to NF-κB as the overexpression of IRAK1 resulted in increased NF-κB activity and the subsequent promotion of tumorigenesis. Conversely, depletion in IRAK1 made cervical cancer cells more vulnerable to radiation [137]. 

A study in glioma demonstrated that high levels of IRAK1 bound to and prevented the degradation of PRDX1, a major member of antioxidant enzymes, in glioma cells, leading to the suppression of autophagic cell death and development of radioresistance. IRAK1 knockdown increased PRDX1 degradation, reduced malignancy, and enhanced radiosensitivity of glioma both *in vitro* and *in vivo* [75].

Recent studies identified a non-canonical IRAK1-induced resistance mechanism to radiation therapy [91,135]. Radiation stimulated the induction of ATR, a DNA damage response kinase, which activates IRAK1 independently from the myddosome complex, thereby facilitating its nuclear translocation through Pellino ligases. Once in the nucleus, IRAK1 acts to inhibit the assembly of a proapoptotic complex, PIDDosome, thereby promoting cell survival and resistance to radiation therapy [91].

Thus, IRAK1 has emerged as a key driver of therapeutic resistance, highlighting the need for increased efforts in developing small-molecule inhibitors and degraders that can effectively target and inhibit its activity, with the goal of preventing or reversing therapeutic resistance.

## 5. IRAK1 Pharmacological Inhibitors

The extensive research on IRAK1 has greatly enhanced our understanding of its role in cancer metastasis and therapeutic resistance, highlighting its clinical relevance as a potential biomarker, therapeutic target, or both, in various solid tumors and hematologic malignancies. IRAK1 could serve as a promising therapeutic target in a large subset of patients harboring IRAK1 gene amplification, protein overexpression, or hyperactivation. Furthermore, the involvement of IRAK1 in therapeutic resistance and radioresistance emphasizes the importance of co-targeting IRAK1 alongside conventional treatments to improve patient outcomes. 

IRAK1 and IRAK4 share several structural similarities, particularly in the ATP-binding pocket where they exhibit >90% similarity [138]. As a result, many inhibitors function as dual IRAK1/IRAK4 inhibitors exhibiting comparable inhibitory potencies. Until recently, most of the IRAK inhibitors used in various research studies to elucidate the biological functions of IRAK1 were, in fact, dual inhibitors of IRAK1 and IRAK4, such as commonly used “IRAK1/4 Inhibitor I” [139]. Significant efforts have been directed towards identifying and developing IRAK1-specific inhibitors or degraders. A summary of selective IRAK1 small-molecule inhibitors is provided in Table 2.

Among the different IRAK1 inhibitors identified, pacritinib is the only one to be tested and used clinically in the US as it was initially recognized as a potent JAK2/FLT3 inhibitor. It was approved in 2022 for the treatment of adults diagnosed with intermediate- or high-risk primary or secondary myelofibrosis, prior to the discovery of its function as an IRAK1 inhibitor [140,141,142,143]. An ongoing phase Ib/II clinical trial (PAIR; NCT04520269) is investigating pacritinib in patients with 1q21.3 amplified solid tumors, which are enriched for IRAK1 pathway activation. One of the trial’s secondary aims–demonstrating pacritinib’s safety in solid tumors with 1q21.3 amplifications—has been achieved. The trial is still ongoing, with the aim of determining the proportion of patients with 1q21.3 amplified breast cancer who remain progression-free 4 months after treatment with pacritinib.

Rosoxacin, a quinolone-derived antibiotic used in the treatment of urinary tract infections and other Gram-negative bacteria, never received approval in the US due to the association of the quinolone class of drugs with long-lasting and disabling side effects, mainly affecting muscles, tendons, bones, and the nervous system [146]. This underscores the need to develop derivatives of rosoxacin that retain IRAK1 selectivity while minimizing toxicities. 

Many *in vitro* and *in vivo* studies have shown that inhibiting IRAK1 presents a promising strategy for the treatment and/or prevention of cancer metastasis and overcoming resistance. However, more work is needed to transition the drugs identified in preclinical studies into clinical trials, as pacritinib remains the only IRAK1 inhibitor currently in clinical use. Additionally, given the relatively recent recognition of the involvement of IRAK1 in tumorigenesis and therapeutic resistance, only a limited number of preclinical studies have explored the potential of combining IRAK1 inhibitors with other treatment approaches to enhance therapeutic efficacies, rather than merely understanding the mechanisms of treatment resistance [75,82,150]. However, with a growing body of evidence elucidating the significant roles IRAK1 plays in tumorigenesis, a significant increase in future studies that utilize IRAK1 inhibitors in combination with conventional treatment modalities is anticipated, aiming to achieve synergistic outcomes in combating cancer metastasis and treatment resistance.

## 6. Conclusions

Increasing evidence suggests that inflammation has been linked to all stages of carcinogenesis, with the TLR/IL-1R axis being a key driver to many inflammatory responses that fuel tumor development, metastasis, and therapeutic resistance. IRAK1 is a crucial downstream mediator of the TLR/IL-1R pathway, significantly contributing to tumorigenesis through the activation of downstream effectors including NF- κB, AP-1, and STAT3. The expression of IRAK1 is elevated in most types of cancer, with notable exceptions in THCA and LAML, highlighting its influence on cancer progression. The involvement of miRNAs, such as miR-146, in regulating IRAK1 expression levels has been extensively studied, highlighting their potential as valuable biomarkers for validating the use of IRAK1-targeted therapies and identifying patients who may benefit from these approaches. Extensive research demonstrated the role of IRAK1 in the various stages of cancer metastasis including tumor cell migration and invasion, angiogenesis, survival, extravasation, metastatic colonization, and priming of the TME by releasing various cytokines and chemokines, all of which help foster conditions that support immune evasion, tumor growth, and survival. Concomitantly with the promotion of metastasis, IRAK1 is also a main contributor to resistance to chemotherapies, targeted therapies, and radiotherapy in various types of cancer. Given the central role IRAK1 has in tumorigenesis and therapeutic resistance, the development of IRAK1-specific small-molecule inhibitors and degraders is critical. Furthermore, the use of combination treatment modalities that incorporate IRAK1-specific inhibitors alongside conventional therapies has become an area of intense focus. These approaches hold the potential to offer synergistic effects, enhancing treatment efficacy and overcoming therapeutic resistance.

## Figures and Tables

**Table 1:** IRAK1 in the multi-step process of metastasis.

Step.	Role of IRAK1	Mechanism Following IRAK1 Activation
Migration and Invasion	Regulation of EMT ^1^	Activation of NLRP3/MAPKs/IL-1β, leading to enhanced migration and invasion [71,72]. Downregulation of epithelial markers and upregulation of mesenchymal markers [62,71,73,74,75]. Activation of NF-κB, leading to acquisition of mesenchymal phenotypes [73].
	Activation of MMPs ^2^	Enhance the expression levels of MMP-2 and MMP-9 through the activation of NF-κB p65 and AP-1 [60,62,76,77,78,79,80,81].
Angiogenesis	Upregulation of pro-angiogenic molecules	Increase the expression of VEGF, CXCL1, and IL-8 [82]. Increase in MVD ^3^ [83].
	Promotion of VSMC proliferation	Activation of ERK1/2, leading to the reduction in p27^Kip1^ and the promotion of proliferation [84].
Survival	Resistance to apoptosis and promotion of tumor growth	Activation of NF-κB, leading to the release of pro-growth cytokines [74,85,86,87,88,89,90]. Activation of mitotic cell cycle and cell division pathways [81]. Resistance of cell apoptosis through nuclear translocation of IRAK1 by Pellino ligases [74,86,87,88,89,90,91].
Extravasation	Promotion of adhesion molecules	Activation of NF-κB, leading to enhanced expression of VCAM-1 and ICAM-1 [92,93,94].
Metastatic Colonization	Immune evasion	Regulation of IFN-γ signaling, leading to a decrease in CD4+/CD8+ T-cell levels [95]. Reduction in the levels of IL-27 in macrophages [96]. Regulation of T-cell targeting chemokine production (CCL5 and CCL20) [97]. Promotion of TAM shift towards M2 polarization [98].
TME	Secretion of pro-inflammatory cytokines	Secretion of pro-inflammatory cytokines such as IL-1β, IL-6, IL-8, IL-10, and TNF-α [99]. Crosstalk between cancer cells and CAF ^4^, leading to the activation of NF-κB and the transcription of *IL-6*, *Cox-2*, *BDNF*, and *IRF-1* [100].

^1^ EMT, epithelial–mesenchymal transition; ^2^ MMP, matrix metalloproteinase; ^3^ MVD, microvessel density; ^4^ CAF, carcinoma-associated fibroblast.

**Table 2:** Selective IRAK1 small-molecule inhibitors and degraders.

Name	Role	Description
Pacritinib (SB1518)	Selective inhibitor	Originally developed as a JAK2/FLT3 inhibitor [140]. Identified as a specific IRAK1 inhibitor through studies conducted in AML [141]. FDA-approved for myelofibrosis [142,143]. Under 26 clinical trials for cancer (ClinicalTrials.gov).
1,4-Naphthoquinone	Selective inhibitor	A quinone-derived compound [144]. Identified as a potent IRAK1 inhibitor through in silico and *in vitro* studies on cancer cells and macrophages [99]. Under preclinical research for the treatment of various types of cancer [145].
Rosoxacin (Acrosoxacin; Eradacil)	Selective inhibitor	A quinolone-derived antibacterial agent [146]. Identified as a specific inhibitor of IRAK1 through studies conducted on autoimmune hypophysitis [146].
JH-X-119-01	Irreversible inhibitor	Developed as a covalent IRAK1 inhibitor and screened on a panel of cancer cells. Activity was confirmed on mutated B-cell lymphoma [87]. THZ-2-118, an IRAK1/4 inhibitor, is the lead compound used for structure development [87]. Currently under preclinical research for lymphoma and sepsis [147,148].
JNJ-1013 (Degrader-3)	Degrader	Developed as an IRAK1 degrader to target IRAK1 scaffolding [149]. Tested on ABC DLBCL cell lines [149].
